# Responsive Feeding, Infant Growth, and Postpartum Depressive Symptoms during 3 Months Postpartum

**DOI:** 10.3390/nu12061766

**Published:** 2020-06-12

**Authors:** Tzu-Ling Chen, Yi-Ying Chen, Chen-Li Lin, Fu-Shiang Peng, Li-Yin Chien

**Affiliations:** 1 Department of Nursing, School of Nursing, National Yang-Ming University, Taipei 11221, Taiwan; zlc1020@gmail.com (T.-L.C.); sthnskimo@yahoo.com.tw (Y.-Y.C.); 2 Department of Nursing, Chang Gung University of Science and Technology, Taoyuan 33303, Taiwan; 3 Department of Obstetrics and Gynecology, Taipei City Hospital, Taipei 10629, Taiwan; DAE34@tpech.gov.tw; 4 Department of Obstetrics and Gynecology, Far Eastern Memorial Hospital, Taipei 22056, Taiwan; knox.p32@msa.hinet.net; 5 Institute of Community Health Care, School of Nursing, National Yang-Ming University, Taipei 11221, Taiwan

**Keywords:** postpartum, responsive feeding, postpartum depressive symptoms, infant growth

## Abstract

Responsive feeding is crucial to the formation of life-long healthy eating behavior. Few studies have examined maternal responsive feeding in early infancy among a Chinese population. This prospective study describes maternal responsive feeding and factors associated with maternal responsive feeding, with emphasis on infant growth and maternal depressive symptoms, during the first 3 months postpartum in Taiwan. From 2015 to 2017, 438 pregnant women were recruited and followed at 1 and 3 months postpartum. Maternal responsive feeding at 3 months was measured on a 10-item 5-point Likert-type scale. Maternal depressive symptoms were measured using the Edinburgh Postnatal Depression Scale with a cutoff score of 10. Infant growth was categorized into four groups based on weight-for-length *Z* scores from birth to 3 months: no change, increase but in the normal range, increase to overweight, and decrease to underweight. Multiple regression revealed that postpartum depressive symptoms, primipara, and decreased infant weight-for-length Z score were negatively associated with maternal responsive feeding, while exclusive breastfeeding and maternal age younger than 29 years were positively associated with maternal responsive feeding. Heath professionals should educate mothers on responsive feeding, with emphases on first-time and non-exclusive breastfeeding mothers, as well as those with depressive symptoms, advanced maternal age, and infants who are becoming underweight.

## 1. Introduction

Responsive feeding is crucial to the formation of life-long healthy eating behavior [1] and is an important protective element for infant health, well-being, and development [2,3]. Most studies on responsive feeding thus far have been conducted in Western cultures [4] and few have examined maternal responsive feeding and associated factors in a Chinese population.

The World Health Organization (WHO) (2009) and UNICEF’s (2016) international standards state that responsive feeding is feeding based on recognizing and appropriately responding to infant needs [5,6]. Previous studies have described two important aspects of low responsive feeding in mother–infant interactions. First, a lack of sensitivity to infant feeding cues characterized by the maternal need to control feeding volume and schedule irrespective of infant hunger or satiation cues. Second, a forced feeding style by which parents use rewards or encouragement to pressure infants into finishing their bottle and/or eating more and show negative emotions if the infants do not do so [7,8,9,10].

Limited instruments assessing maternal responsive feeding are intended for infants younger than 6 months [11]. Recently, Sall et al. (2020) designed a responsive feeding scale for children 6–23 months old. Though the scale showed acceptable reliability and validity in Cambodian children [3], use of the scale among infants younger than 6 months in a high-income Asian country, such as Taiwan, needs further validation. To the best of our knowledge, none of the responsive feeding scales have been validated in Taiwan. A study in urban New York found that most mothers wrongfully believed that infant crying and hand sucking were hunger cues, while over half believed that infants should finish their bottles [9]. An Australian study reported that scheduled feeding (only feeding at scheduled times) was positively associated with rapid weight gain (>0.67 weight-for-age Z score) from birth to 4–7 months [12]. A cohort study found that lower maternal sensitivity to infant cues at 6 months significantly predicted higher infant weight gain between 6–12 months [13]. Contrastingly, another study found that when maternal control over feeding was low or moderate, infants were able to self-regulate their weight during their first year, such that infants that showed slow early weight gain from birth to 6 months accelerated in their subsequent weight gain from 6 to 12 months, while those with greater early weight gain decelerated. When maternal control in feeding was high, weight gain in the first year of life was consistently higher [14]. Fuglestad et al. (2017) found that maternal use of infant based hunger cues at 3 months in their feeding practices were associated with lower gains in weight-for-length and body fat between 2 weeks and 3 months among infants [15]. As such, the literature appears to support the presence of an association between higher infant weight gain and low responsive feeding.

The prevalence of postpartum depressive symptoms in Taiwan was higher than 20% [16]. Depressive symptoms among mothers could block a mother’s attention to her infant’s cues for feeding and influence her sensibility to respond. Depressed mothers are less sensitive to infant needs during early infancy [17,18]. In a large sample of Australian first-time mothers of infants aged 2–7 months, mothers with higher depression scores were more likely to use food to calm infants, less aware of infant cues, and more concerned over their child’s intake and weight [8,10]. Another study in the United States showed that depressed mothers were positively associated with using forceful feeding styles from birth to 12 months [19].

In terms of other factors associated with responsive feeding, a study found that increased maternal age and higher educational level were associated with a more controlling feeding style of the parents [20]. Another study reported that responsive feeding was positively associated with maternal education, breastfeeding, and female infant gender [19]. First time mothers could be less sensitive to infant cues and thus decrease the quality of feeding practices [21]. Mothers of infants whose birth gestational age was less than 32 weeks were more likely to use food rewards or encourage more intake [22]. Therefore, those variables were included in the study.

The review of literature shows that the effect of infant growth status and maternal postpartum depressive symptoms on responsive feeding is less studied in a non-Western population, particularly among infants younger than 3 months. The study aimed to (1) examine maternal depression and infant growth status as predictors of maternal responsive feeding; (2) explore other factors, including socio-demographics, breastfeeding, pre-pregnancy body mass index (BMI), infant characteristics, and obstetrical variables associated with maternal responsive feeding at 3 months postpartum in Northern Taiwan. We hypothesized that the presence of maternal depression and increased infant growth are associated with a lower level of maternal responsive feeding.

## 2. Materials and Methods

### 2.1. Sample and Setting

This study is part of a larger study aiming to examine maternal and child weight trajectory from pregnancy to 18 months postpartum, which formed the basis for the targeted sample size. We recruited 800 pregnant women during their second trimester of pregnancy (14–27 weeks). The study participants were recruited from 4 hospitals and 2 clinics in Taipei, Taiwan. Eligible participants were pregnant women aged ≥20 years, who were 14–27 weeks pregnant, without major maternal morbidity at the time of recruitment, singleton pregnancy, and were able to complete the questionnaires in Mandarin Chinese. Women who agreed to participate in this study signed a consent form and filled out the structured questionnaire during the second trimester in the antenatal visits and were followed at the third trimester, 1 month, and 3 months postpartum. The study protocol was approved by the institutional review board at the National Yang-Ming University, Taipei City Hospital, Far Eastern Memorial Hospital, Taipei Tzu Chi Hospital, and Taiwan Adventist Hospital.

### 2.2. Sample Size Consideration

To build a predictive model for responsive feeding, when the effect size was moderate (R^2^ = 0.13), power = 0.80, alpha = 0.05, and 10 predictors in multiple regression, the required sample size was 119 [23]. The current sample size is deemed to be acceptable for this analysis.

### 2.3. Measures

The variables measured at each time point are presented in Table 1. Besides responsive feeding, maternal depressive symptoms, and infant growth, the study variables included socio-demographics (maternal age and family socio-economic status), breastfeeding, pre-pregnancy BMI, infant characteristics (sex and gestational age), and obstetrical variables (delivery mode and parity). The family socio-economic status were classified into high (41–55), middle (30–40), and low (11–29) groups based on the Hollingshead (1957) 2-factor index of social position [24], which was later adapted to Taiwan by Lin (2005) [25]. The maternal body weights and heights were based on self-report and the pre-pregnancy BMI was calculated by the researcher. BMI cut-off based on CDC (2016) guidelines was used to classify participants as underweight (<18.5 kg/m^2^), normal weight (18.5–24.9 kg/m^2^), overweight (25.0–29.9 kg/m^2^), and obese (≥30 kg/m^2^) [26]. Breastfeeding status was classified into 3 categories: exclusive breastfeeding, partial breastfeeding, and formula feeding. Exclusive breastfeeding consisted of the subset of infants who were fed only breast milk, with no supplemental formula used. Partial breastfeeding consisted of the subset of infants who were fed breast milk and supplemental formula to any degree. Formula feeding consisted of the subset of infants who were fed only formula. None of the study participants reported adding complementary food at the 1- and 3-month questionnaire. Maternal depressive symptoms were measured with the Chinese version of the Edinburgh Postnatal Depression Scale (EPDS), which consists of 10-items on a 4-point Likert scale with a cut-off score of 10 (EPDS >10) for depression symptoms. Previous studies reported that the cut-off score of 10 performed substantially better in distinguishing depressed from non-depressed Taiwanese postpartum women [27]. In this study, the Cronbach’s alpha was 0.89 at 1 month postpartum.

Infant body weight and length at birth and at 3 months was from the Child Health Manual, which was recorded by doctors and nurses during clinic visits. Weight-for-length Z score (WLZ) was calculated based on the WHO infant growth chart calculator (0–3 years) of <−2 standard deviation (SD) (underweight), −2 to 2 SD (normal), and >2 SD (overweight) by infant gender (WHO Anthro version; WHO, 2013) [28]. Although the WHO growth curve was based on breastfeeding infants, the CDC and WHO recommend that the growth curve is applicable to infants ≤24 months, regardless of their breastfeeding status [29]. WLZ scores for infants at birth to 3 months could have 9 combinations, however we could only have 4 categories after accounting for missing and loss to follow up. Therefore, infant at-birth to 3 months WLZ scores were categorized into 4 groups as follows: no change (WLZ is normal at birth and 3 months), increase but in normal range group (WLZ is underweight at birth and normal at 3 months), increase to overweight group (WLZ is normal at birth and overweight at 3 months), and decrease to underweight group (WLZ is normal at birth and underweight at 3 months).

Responsive feeding among mothers was measured using a scale developed for this study through self-report (items are listed in Table 2). We defined responsive feeding as recognizing and responding to the signs the baby gives about whether he or she is hungry or full [30], which included two dimensions—feeding on demand and unforced feeding [7,10,13,31,32]. Feeding on demand refers to a feeding schedule and volume depending on the infant’s demand rather than maternal control. Unforced feeding refers to not pressuring infants to eat and showing negative emotions when the infant cannot finish. The scale included 10 items rated on a 5-point Likert scale, with a possible range from 0 to 40. Of the 10 items (Table 2), item 7 and 10 were coded as 0: never, 1: seldom, 2: sometimes, 3: frequently, 4: always, and the remaining 8 items were coded as 4: never, 3: seldom, 2: sometimes, 1: frequently, 0: always). A higher score indicated higher responsive feeding. In Table 2, the first 6 questions are for unforced feeding and the rest of the 4 questions are for feeding on demand.

The content validity of the responsive feeding scale was assessed by five experts in maternal and child health. The experts rated the correctness, appropriateness, and clarity of the questionnaire using a 4-point Likert scale ranging from 1 (very inadequate) to 4 (very adequate). Items that had low scores (<3) were discussed and modified by the experts. Content validity index, as determined by the number of experts giving a rating of 3 or 4, divided by the number of experts, then averaging the item-level results [23], was 0.98. The questionnaires were pilot tested with 10 postpartum mothers to ensure semantic clarity and readability. Exploratory factor analysis using principal components following varimax rotation with Kaiser normalization was conducted on the responsive feeding scale. Initial factors were determined by factors with an eigenvalue greater than 1 and examination of the scree plot. The results demonstrated the 2-factor structure of the scale with item factor loadings all above 0.3. The explained variance of the responsive feeding scales was 44.6% (Table 2). The results of factor analysis supported the construct validity of the scale. The internal reliability of the scale was average (Cronbach’s alpha = 0.65 for the total scale; 0.69 for unforced feeding; and 0.60 for feeding on demand). The correlation between the two subscales was 0.2 (*p* < 0.001). We regard the correlation as acceptable.

### 2.4. Study Design and Procedure

This study was a prospective cohort study. Recruited participants filled in a structured questionnaire during their antenatal visits in their second trimester. Participants were also followed up during their third trimester and their 1st and 3rd month postpartum. Participants received a phone call a week prior to the designated time and selected their preferred method of filling in the structured questionnaire, including telephone interview, post mail, and email. Follow-up phone calls were made if the questionnaire was not returned within 2 weeks. Data were collected from March 2015 through May 2017.

### 2.5. Statistical Analysis

The data analysis was performed using SPSS for Windows version 21.0 (SPSS Inc., Chicago, IL, USA). The descriptive statistics used were mean, SD, frequency, and percentage depending on the type of variables. We drew a crude X-Y plot and visually determined whether the association was linear. If not, the variable was categorized into categories based on previous literatures and/or using the data to find the cut-off for showing differences. Bivariate analysis was examined using t-test or ANOVA. Multivariate linear regression analyses were performed using the responsive feeding scale score as the dependent variable. We first started by fitting all included variables and then dropped one least significant variable at a time until all variables in the linear regression model were statistically significant. We checked normality of the residuals using a normal predicted probability (P-P) plot. To account for multiple comparisons and the exploratory nature of finding associated factors of responsive feeding, the significance level was set at *p* value < 0.01 [33,34]. The bi-variate analysis showed a crude association between X (independent variables) and Y (dependent variable). The multi-variate analysis not only showed an adjusted association between X and Y, but also built a predictive model. Aside from the primary data analysis, we performed multiple imputation to deal with the missing data [35]. Multiple imputation of the final multiple regression analysis appeared to be similar to the results from the primary data analysis. Therefore, we only presented data analysis of those with completed data in the paper.

## 3. Results

### 3.1. Sample Flows

**Figure 1:**
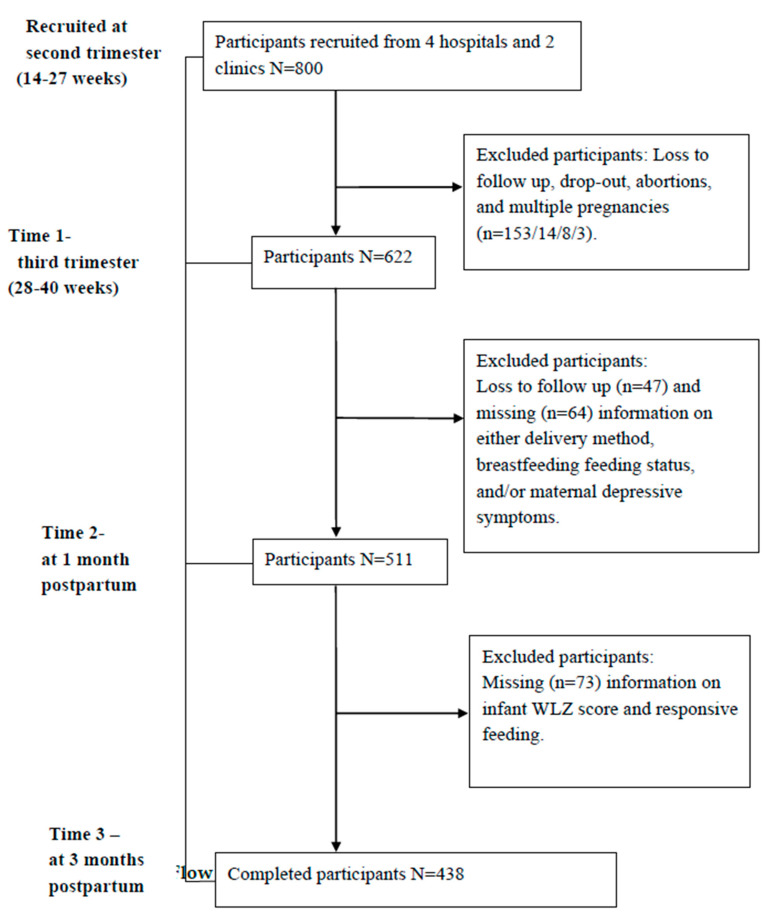
Flow diagram of the sample.

Of the 800 recruited women at the second trimester, 622 women completed the questionnaire at the third trimester. A total of 511 (82.1%) and 438 (70.4%) women completed the questionnaire at 1 and 3 months after delivery, respectively. This analysis included the 438 women who completed all the three follow-up questionnaires (Figure 1). We compared the characteristics of the women who completed all three follow-up questionnaires (*n* = 438) to those who completed the first questionnaire only (*n* = 800). There were no significant differences in age, parity, maternal work status, socio-economic status, breastfeeding status, or pre-pregnancy BMI between the two groups.

### 3.2. Participant Characteristics

Participant characteristics are presented in Table 3. The mean age of the participants was 33.3 (range, 20–43) years. Most participants reported an educational level of university or higher (87.7%). Approximately 15% of the women were overweight or obese, while 16% were underweight before pregnancy. Approximately 56.4% of participants were primipara and 31.3% had a cesarean delivery. The prevalence of exclusive breastfeeding at 1 month postpartum was 44.7%. The prevalence of depressive symptoms at 1 month postpartum as determined by EPDS score ≥10 was 27.9%. The prevalence of underweight, normal, and overweight among infants were 5.0%, 94.7%, and 0.2%, respectively, at birth and were 3.2%, 90.2%, and 6.6%, respectively, at 3 months postpartum.

### 3.3. Descriptive of the Responsive Feeding Scale

The mean responsive feeding score among mothers was 28.73 ± 5.89 (range, 11–40). A medium level of responsive feeding was reported by our study participants and the item with the lowest score was “You only feed the baby at the fixed time” (Table 2). Other items that had a mean score of <3 were “You would let the baby decide when to eat and finish,” “If the baby did not eat enough, you would feel depressed,” “Even if the baby is not hungry, you will feed him/her according to the scheduled time,” “You worry about the baby not eating enough,” and “Whenever the baby wants to eat, you feed him/her.”

### 3.4. Bi-Variate Analysis on Factors Associated with Responsive Feeding

The mean responsive feeding scores by characteristic are presented in Table 4. Mean responsive feeding scores differed significantly by maternal age, parity, breastfeeding at 1 month, and infant weight growth. Mothers whose age was ≤29 had higher responsive feeding scores (30.62 ± 5.15) than 30–34 years (28.35 ± 5.76) and ≥35 years (28.41 ± 6.18) mothers. Primipara mothers had lower mean responsive feeding scores than multipara mothers (27.84 ± 5.59 vs. 29.89 ± 6.07). The responsive feeding score was significantly higher among mothers that exclusively breastfed their infants at 1 month postpartum (30.30 ± 5.71) than those that partially breastfed (27.60 ± 5.79) and those that formula fed (26.96 ± 5.50). The responsive feeding score was significantly lower for infants in the decreased to underweight (23.13 ± 6.12) than the other three groups (mean score ranged 28.41–30.90).

### 3.5. Multi-Variate Results on Factors Associated with Responsive Feeding

The multiple linear regression model (Table 5) showed that postpartum depressive symptoms at 1 month postpartum (β = −0.14, *p* < 0.01) and primipara (β = −0.14, *p* < 0.01) were negatively associated with responsive feeding. WLZ scores for infant at-birth to 3 months in the decrease to underweight group (β = −0.12, *p* < 0.01) were also negatively associated with responsive feeding. Then, maternal age ≤29 years (β = 0.16, *p* < 0.01) and exclusive breastfeeding at 1 month postpartum (β = 0.22, *p* < 0.01) were positively associated with responsive feeding. The model explained approximately 14% (adjusted *r*^2^ = 0.13) of the variance in the responsive feeding scores and was statistically significant (*F* = 8.00, *p* < 0.001).

## 4. Discussion

We found that infants with WLZ scores in the decrease to underweight group between birth to 3-months reported significantly lower responsive feeding scores relative to the increase or no change groups. It is noted that our decrease to underweight group was infants whose WLZ was normal at birth and underweight at 3 months, which was different from growth status measured in previous studies. In addition, measurement of responsive feeding differed across studies and we cannot find studies that relate growth status from birth to 3 months to responsive feeding at 3 months. Therefore, our results are not directly comparable to previous studies. Nonetheless, our result is in line with a study of African American babies between 3 and 18 months, which reported pressuring feeding, as characterized by the parent increasing the amount of food the infant consumes (conceptually similar to subscale “unforced feeding” in this study), which was associated with lower infant weight-for-age [36]. However, our finding is different from a study showing that maternal feeding decision based on infant cues (conceptually similar to the subscale “feeding on demand” in this study) at 3 months was associated with a slower growth in WLZ between 2 weeks and 3 months [15]. The direction of association between infant growth status and responsive feeding merits further study.

A previous UK study found that mothers of infants with lower birth weight (birthweight standard deviation score < −0.56) or lower appetite or that were more concerned about their infant being underweight during the first 3 months were more likely to use pressure while feeding at 8 months. Feeding pressure was measured by the item “have a break then try again/encourage to eat/feed sooner/take a larger amount” [37]. The items were conceptually similar to the items in subscale “unforced feeding” in this study. We did not find that lower birthweight or gestational age was associated with responsive feeding in our study, but we found that decrease to underweight from birth to 3 months was negatively associated with responsive feeding. Altogether, the results suggested that infant birth weight or growth status was associated with early feeding behavior. Decrease to underweight in early life or lower birth weight could cause maternal concern and negatively influence responsive feeding. Furthermore, low responsive feeding in early life could later lead to obesity [38]. Further studies are needed to examine the reciprocal relationship between child growth and maternal feeding practices. Nonetheless, when providing guidance on infant feeding, health professionals should be aware of the growth status of the respective infants, assess parental concerns about infant growth, and support parents to establish feeding behavior based on the infants’ demands and responses to ensure optimal feeding outcomes.

The mean responsive feeding score was found to be moderate (71.8/100) and the item with the lowest score was “You only feed the baby at a fixed time.” This is perhaps in line with a previous finding that indicated that Chinese immigrant mothers believe in the importance of formulating and leading eating schedules and routines for preschoolers [39]. The items with the lowest scores (<3) in the present study were across the two main concepts, namely unforced feeding and feeding on demand. Thus, unforced feeding and feeding on demand should both be accentuated in the future design of parental health education.

In the present study, the prevalence of postpartum depression symptoms as determined by EPDS ≥10 among mothers at one month postpartum was 27.9%. It was noted that none of the mothers received a diagnosis of depression or took drugs at that time. Therefore, the results may not be generalized to mothers with diagnosed depression or more severe depression. Reviews showed that the prevalence of postpartum depression symptoms ranged widely from 4% to 69% globally and ranged from 27.4% to 31.6% in China and 23% to 24.1% in Taiwan [16]. In addition to high postpartum depression symptoms, we further found that depressed mothers were significantly associated with less responsive feeding practices. Comparable to the present findings, a previous study found that depressed mothers were more likely to add cereal to the bottled infant formula to feed their infants at 2 months [40], though such behavior was not reported in the current study. These findings underscore the need to screen mothers with postpartum depression symptoms, refer them to psychiatric services if needed, and provide education and support for these mothers while they feed their infants.

Our results found that exclusive breastfeeding at 1 month postpartum was a significant predictor of responsive feeding at 3 months postpartum, which is consistent with prior findings [41]. Breastfeeding is typically baby-led and milk lactation is usually based on infant sucking and demand, whereas formula feeding can be controlled by parents that use fixed feeding schedules and quantities [20,41,42]. Therefore, breastfeeding mothers were more likely to engage in voluntary feeding and feed on infant demand. Health professionals could teach formula-feeding mothers how to recognize infant hunger/satiety cues and thus avoid overfeeding.

We found that primiparous and older mothers each were independently associated with lower levels of responsive feeding. A previous study showed that maternal age was positively associated with parent-led routine [20], which would be classified as low responsive feeding in this study. Perhaps, this is because first-time mothers may feel less confident in their capacity to satisfy their infant’s needs [43], while older mothers are more likely to use parent-led routines in feeding the infant [20]. Health professionals should pay particular attention and assess feeding practices among such mothers and guide them if necessary.

Responsive feeding measure was specifically developed for this study. The scale demonstrated acceptable construct validity and internal consistency in terms of the two subscales, unforced feeding and feeding on demand, composing the responsive feeding scale. Previous studies have supported that feeding on demand and unforced feeding are two important aspects of responsive feeding [7,10,13,31,32]. We decide to use the full scale score rather than the sub-scale score since the full score has more variability, owing to its higher number of items than the subscale score. However, responsive feeding is a multi-dimensional concept and was measured differently across studies [3,4]. Some dimensions are not included in the current scale, for example, indulgent feeding, maternal sensitivity to infant cues, and uninvolved feeding. In addition, this study focused specifically on the reciprocal feeding relationship between infants and their mothers. Yet prior studies have indicated that the family and environment also represent important determinants in the formation of child eating behavior [3,44,45]. Future studies could expand and incorporate more dimensions such as environmental and familial factors in the measurement of responsive feeding. In addition, future longitudinal developmental studies observing children from infancy to preschool should be conducted to examine eating behavior over time and attempt to link early feeding behavior to eating behavior in children.

Infant growth was categorized into four groups rather than a simple WLZ difference in the study. We drew the crude X-Y plot for changes in WLZ and responsive feeding and found that the association was not linear and the pattern was not clear. Given that the definition of normal, underweight, and overweight is widely used in determining infant body size, we decided to use the classification at the two time points (birth and 3 months) to divide infant growth into categories. Further studies are needed to validate optimal ways to classify infant growth.

The present study results are exploratory in nature. The significant results presented in bi-variate and multi-variate analysis are statistically significant. Whether the statistically significant results mean clinical significance merits further study. The fact that certain characteristics have a small sample size suggests the need to replicate the findings in future studies in order to further validate the results. The explanatory power of the multiple linear regression model in the prediction for responsive feeding does not seem high (r^2^ = 0.14), yet this is similar to findings of previous studies [46] and is deemed acceptable [23,47].

This study focused on maternal feeding behavior and feeding behavior of other caregivers is not considered. Though mothers play a central role in early infant feeding, other caregivers may also play a role, especially for formula-fed babies. Future studies should take other caregivers into account in order to have a more complete picture. Type of food consumed by mothers or type of formula chosen for babies could influence infant growth, but whether those would influence responsive feeding merits further study.

This study has a number of limitations. First, responsive feeding was measured based on maternal self-report, yet mothers may respond in a socially desirable way and objective validation of their veracity of their reports was not possible. Second, the internal consistency of the responsive feeding scale was assessed as a Cronbach’s alpha value of 0.65 and further validation of the scale may be needed. The correlation between current measure and existing validated responsive feeding scale that used observation during feeding should be examined in future studies to further assess the scale validity. Since the study used a self-developed measure for responsive feeding, the comparability of the study results to previous findings are limited. Infant weight and length were measured by doctors and nurses in the clinics and obtained from the Child Health Manual. We assume that the infant anthropometric measures in the clinics are acceptable, but we did not verify the reliability and validity of the measures. We successfully followed 438/800 or 55% of the recruited pregnant women at three months postpartum. We compared the results from the primary data analysis to the analysis after multiple imputation and found the estimates to be similar. Nonetheless, such loss to follow-up could pose a threat to the internal validity. Our study mothers appear to be older and have a high educational level, and only a few study infants are outside of the normal WLZ ranges when compared to samples in previous studies. However, the distribution in those characteristics is not very different from other studies conducted in Taipei, the capital of Taiwan. The prevalence of 15.1% for overweight/obese pre-pregnant women and 44.7% for exclusive breastfeeding at 1 month postpartum in the study is similar to those in previous studies [48,49,50], suggesting generalizability of the findings. The associations reported in the current study were correlational and given the measurement and analysis performed, future longitudinal studies with a longer period of follow-up are needed to establish the causal relationship.

## 5. Conclusions

This study found that decreased infant weight-for-length Z score from birth to 3 months, maternal postpartum depressive symptoms, and primipara were negatively associated with responsive feeding. Contrastingly, exclusive breastfeeding during the first month of life and maternal age younger than 29 years were positively associated with responsive feeding. The study results supported that early infant growth and maternal postpartum depressive symptoms are associated with infant feeding behavior in a Chinese population. Health professionals should educate mothers on responsive feeding and place a particular emphasis on first-time, older, and non-exclusive breastfeeding mothers. Care should also be given to mothers exhibiting depressive symptoms and infants becoming underweight. During children’s clinical visits, health professionals should assess as well as potentially track infant growth and suggest interventions accordingly. Thus, overall, this study indicates that further attempts should be made to boost maternal support and confidence to encourage mothers to perform responsive feeding.

## Figures and Tables

**Table 1:** Study variables and their time point of data collection.

Measurement Time Points	Variables
During pregnancy	Age, educational level, family socio-economic status, parity, and maternal pre-pregnancy body mass index
1-month postpartum	Gestational age at birth, mode of delivery, breastfeeding status, maternal depressive symptoms, infant gender, birth weight, and birth length
3-month postpartum	Infant body weight and length at 3 months, responsive feeding

**Table 2:** Exploratory factor analysis of the responsive feeding scale at 3 months postpartum (*n* = 438).

Items	Mean ± SD	Factor Loading	
		Unforced Feeding	Feeding on Demand
1. If the baby did not eat enough, you would feel depressed ^a^	2.60 ± 1.32	0.82	0.04
2. Worry about the baby not eating enough ^a^	2.84 ± 1.24	0.76	−0.10
3. When you see other babies at the same age weighing more than your baby, you feel that your baby’s weighing less is your responsibility ^a^	3.40 ± 1.05	0.66	−0.01
4. Feeding is like a fight ^a^	3.31 ± 0.94	0.58	0.04
5. When the baby is not willing to eat, you still continue feeding ^a^	3.19 ± 1.00	0.45	0.33
6. You will feed more to make sure the baby is full ^a^	3.45 ± 0.91	0.32	−0.05
7. Whenever the baby wants to eat, you feed him/her	2.91 ± 1.25	−0.13	0.72
8. You only feed the baby at the fixed time ^a^	1.75 ± 1.46	−0.02	0.75
9. Even if the baby is not hungry, you will feed him/her according to the scheduled time ^a^	2.69 ± 1.34	0.43	0.61
10. You would let the baby decide when to eat and finish	2.59 ± 1.41	−0.06	0.60
Eigenvalue		2.52	1.94
% of variance		25.19	19.41

Note: ^a^ indicated reversely coded items. Higher scores indicate higher levels of responsive feeding.

**Table 3:** Participant characteristics (*n* = 438).

Variable	M ± SD or *n* (%)
Maternal age (years)	**33.3 ± 4.1**
≤29	69 (15.8%)
30–34	195 (44.5%)
≥35	174 (39.7%)
Spousal age (years)	**35.7 ± 10.8**
≤29	52 (11.9%)
30–40	361 (82.4%)
41–50	25 (5.7%)
Educational level	
Senior high school or lower	54 (12.3%)
University or higher	384 (87.7%)
Family socio-economic status	
Low	77 (17.6%)
Middle	187 (42.7%)
High	174 (39.7%)
Pre-pregnancy BMI	
Underweight	70 (16.0%)
Normal	302 (68.9%)
Overweight	49 (11.2%)
Obesity	17 (3.9%)
Parity	
Primipara	247 (56.4%)
Multipara	191 (43.6%)
Delivery method	
Vaginal delivery	301 (68.7%)
Cesarean delivery	137 (31.3%)
Gestational age at birth	
32–36 weeks	23 (5.3%)
≥37 weeks	415 (94.7%)
Breastfeeding status at 1 month postpartum	
Exclusive breastfeeding	196 (44.7%)
Partial breastfeeding	190 (43.4%)
Formula feeding	52 (11.9%)
Maternal depressive symptoms at 1 month postpartum	
No	316 (72.1%)
Yes	122 (27.9%)
Infant gender	
Male	236 (53.9%)
Female	202 (46.1%)
Infant weight-for-length at birth	
Underweight	22 (5.0%)
Normal	415 (94.7%)
Overweight	1 (0.2%)
Infant weight-for-length at 3 months	
Underweight	14 (3.2%)
Normal	395 (90.2%)
Overweight	29 (6.6%)
Infant at-birth to 3 months WLZ scores	
No change	372 (84.9%)
Increase but in normal range	22 (5.0%)
Increase to overweight	29 (6.6%)
Decrease to underweight	15 (3.4%)

**Table 4:** Mean responsive feeding scores by participants’ characteristics (*n* = 438).

Variables	Mean (SD)	t/F	*p*	Post Hoc Tests
Maternal ages (years)		4.29 *	0.01	“≤29” > “30–34” *
≤29	30.62 (5.15)			“≤29” > “≥35” *
30–34	28.35 (5.76)			
≥35	28.41 (6.18)			
Family socio-economic status		1.98	0.14	-
Low	29.86 (5.22)			
Middle	28.71 (6.08)			
High	28.26 (5.91)			
Parity		3.67 *	<0.01 *	-
Primipara	27.84 (5.59)			
Multipara	29.89 (6.07)			
Pre-pregnancy BMI		1.41	0.24	-
Underweight	27.67 (6.06)			
Normal	28.80 (5.94)			
Overweight	29.20 (5.12)			
Obesity	30.53 (5.96)			
Delivery method		−0.31	0.76	-
Vaginal birth	28.67 (5.79)			
Cesarean birth	28.86 (6.11)			
Gestational age at birth			0.14	-
32–36 weeks	26.96 (4.28)	−1.49		
≥37 weeks	28.83 (5.95)			
Breastfeeding status at 1 month postpartum		13.57 *	<0.01 *	Exclusive > Partial * Exclusive > Formula *
Exclusive breastfeeding	30.30 (5.71)			
Partial breastfeeding	27.60 (5.79)			
Formula	26.96 (5.50)			
Maternal depressive symptoms at 1 month postpartum		3.91 *	<0.01 *	-
Yes	26.99 (5.95)			
No	29.41 (5.73)			
Infant gender		0.61	0.14	-
Male	28.35 (5.93)			
Female	29.18 (5.82)			
Infant growth status from birth to 3 months		6.08 *	<0.01 *	I > IV * II > IV *
No change (I)	28.81 (5.81)			III > IV *
Increase but in normal range (II)	28.41 (5.36)			
Increase to overweight (III)	30.90 (5.56)			
Decrease to underweight (IV)	23.13 (6.12)			

Note: * *p*< 0.01, - indicates that no post-hoc test was performed.

**Table 5:** Multiple linear regression model for responsive feeding at 3 months postpartum.

Variables	β (95% CI)	*p* Value
Maternal age (years)		
≤29	0.16 (1.99, 4.14)	<0.01 *
30–34	0.01 (−1.05, 1.26)	0.86
≥35	1	
Parity		
Primipara	−0.14 (−2.75, −0.57)	<0.01 *
Multipara	1	
Breastfeeding status at 1 month postpartum		
Excusive breastfeeding	0.22 (0.84, 4.29)	<0.01 *
Partial breastfeeding	0.04 (−1.24, 2.19)	0.58
Formula feeding	1	
Maternal depressive symptoms at 1 month postpartum	−0.14 (−3.04, −0.69)	<0.01 *
Infant growth—birth to 3 months		
No change	1	
Increase but in normal range	−0.01 (−2.74, 2.01)	0.76
Increase to overweight	0.08 (−0.24, 3.95)	0.08
Decrease to underweight	−0.12 (−7.10, −1.18)	<0.01 *

Note: * *p* < 0.01. The model R^2^ = 0.14, adjusted R^2^ = 0.13, SSE = 5.50.
